# Learning State-Variable Relationships in POMCP: A Framework for Mobile Robots

**DOI:** 10.3389/frobt.2022.819107

**Published:** 2022-07-19

**Authors:** Maddalena Zuccotto, Marco Piccinelli, Alberto Castellini, Enrico Marchesini, Alessandro Farinelli

**Affiliations:** Department of Computer Science, University of Verona, Verona, Italy

**Keywords:** planning under uncertainty, POMCP, POMDP, prior knowledge, Markov Random Fields, learning, mobile robot planning

## Abstract

We address the problem of learning relationships on state variables in Partially Observable Markov Decision Processes (POMDPs) to improve planning performance. Specifically, we focus on Partially Observable Monte Carlo Planning (POMCP) and represent the acquired knowledge with a Markov Random Field (MRF). We propose, in particular, a method for learning these relationships on a robot as POMCP is used to plan future actions. Then, we present an algorithm that deals with cases in which the MRF is used on episodes having unlikely states with respect to the equality relationships represented by the MRF. Our approach acquires information from the agent’s action outcomes to adapt online the MRF if a mismatch is detected between the MRF and the true state. We test this technique on two domains, rocksample, a standard rover exploration task, and a problem of velocity regulation in industrial mobile robotic platforms, showing that the MRF adaptation algorithm improves the planning performance with respect to the standard approach, which does not adapt the MRF online. Finally, a ROS-based architecture is proposed, which allows running the MRF learning, the MRF adaptation, and MRF usage in POMCP on real robotic platforms. In this case, we successfully tested the architecture on a Gazebo simulator of rocksample. A video of the experiments is available in the Supplementary Material, and the code of the ROS-based architecture is available online.

## 1 Introduction

Planning under uncertainty is a problem of sequential decision-making, which has important applications in artificial intelligence and robotics. Over the last 2 decades, the interest in this topic has grown rapidly due to methodological improvements and the application of these techniques to real-world domains, such as smart buildings, industrial machinery controllers, and mobile robot navigation. Intelligent and autonomous agents have been, in fact, recently employed in complex domains (e.g., search and rescue, warehouse pick-and-place operations, and mobile robot navigation) where the environment is only partially observable. In such domains, it is hard to have complete knowledge of the environment in which the agent acts. In this work, we tackle a specific problem in the context of planning under uncertainty: the problem of learning probabilistic state-variable relationships. Consider, for instance, a warehouse made of aisles with different traffic levels. A robot has to move in the warehouse to accomplish some tasks. The state of the system contains the robot’s position and the configuration of traffic levels in each aisle, but the traffic levels are not known by the robot. It has to discover them using noisy sensors while moving in the warehouse. In this case, each state variable represents the traffic level of an aisle. In the following, state variables refer to the hidden part of the state. Learning state-variable relationships in this context means learning the relationships among the traffic levels of different aisles in the warehouse. The rationale is that if the robot knows that two aisles have the same traffic levels with a high probability, it can improve its planning performance because once it has observed the traffic level of one aisle, it has also acquired some knowledge about the traffic of the other aisle and it can plan considering that knowledge.

Partially Observable Markov Decision Processes (POMDPs) (Sondik, 1978; Kaelbling et al., 1998) are a powerful framework for planning under uncertainty. Markov Decision Processes (MDPs) (Russell and Norvig, 2010) are extended to the case of partially observable environments. To tackle partial observability, they consider all possible states of the (agent-environment) system and assign to each of them a probability value expressing the related likelihood of being the true state. These probabilities, considered as a whole, constitute a probability distribution over states, called belief. A solution for a POMDP is a policy that maps beliefs into actions. The computation of optimal policies is unfeasible in practice (Papadimitriou and Tsitsiklis, 1987). Therefore, much effort was put into developing approximate (Hauskrecht, 2000) and online (Ross et al., 2008) solvers. The most recent approaches mainly rely on the use of point-based value iteration (Spaan and Vlassis, 2004, 2005; Veiga et al., 2014) or Monte-Carlo Tree Search (MCTS) based solvers (Kocsis and Szepesvári, 2006; Browne et al., 2012) to deal with large state spaces. Deep Reinforcement Learning (DRL) approaches are instead used to learn policies directly from observations, without using a model of the environment dynamics (Silver et al., 2016, 2017; Sutton and Barto, 2018). Planning and reinforcement learning methods have also been used together (Leonetti et al., 2016) to allow adaptation to the environment and increased reliability. Among the main MCTS-based solvers (Thrun, 2000; Kocsis and Szepesvári, 2006), a meaningful improvement was obtained by Partially Observable Monte Carlo Planning (POMCP) (Silver and Veness, 2010), a pioneering algorithm that allows applying model-based reinforcement learning to very large state spaces, overcoming the scalability problem that has limited the usage of POMDPs for many years.

We apply the proposed approach for learning state-variable relationships to POMCP. The standard version of this algorithm does not consider any kind of prior knowledge about state-variable relationships. Castellini et al. (2019) proposed an extension of POMCP, which considers these relationships in the form of Constraint Networks (CNs) or Markov Random Fields (MRFs). In that work, the introduction of such knowledge provides an improvement in terms of planning performance, with no additional overhead in terms of time complexity. However, it is assumed to have full knowledge about the CN or MRF containing the state-variable constraints. This knowledge could be provided, for instance, by experts. Herein, instead, we deal with a methodology for learning this knowledge in the form of an MRF. The literature provides some general approaches for learning MRFs, mainly in the context of computer vision (Vuffray et al., 2020; Shah et al., 2021), but they are very general and often time-consuming. On the contrary, our proposed approach is specialized in planning under uncertainty with POMDPs. Hence, it integrates with POMCP without increasing its time complexity. Learning pairwise MRFs, instead of general MRFs, requires a smaller amount of data, which is important in the context of planning, where each learning episode can take a long time. Let us consider, for instance, the case study of the warehouse mentioned above, in which a learning episode could last an entire day of work, having the autonomous robot collect data about traffic levels in the aisles while doing its job.

In this work, we propose three methodological advancements. The first is an algorithm for learning the MRF during the execution of POMCP. The second is implementing a framework to integrate POMCP in ROS, enabling the employment of the MRF learning algorithm on real robotic platforms, with experiments performed on Gazebo simulators of known application domains. The ROS-based architecture allows learning the MRF on real robotic platforms. It comprises three ROS nodes: environment, agent, and planning. The environment node discretizes the real world by exploiting a task-specific representation. The agent node, instead, holds information about odometry and interfaces the ROS-based robotic platform with the environment and the planner. Finally, the planner node runs the learning algorithm. The third advancement is an algorithm called “Adapt” (see Algorithm 2), which deals with cases in which we use the learned MRF in episodes with unlikely state-variable configurations with respect to the joint probability defined by MRF. This algorithm runs when the knowledge provided by the learned MRF does not reflect the true state-variable values. In such cases, the MRF is misleading because it forces the belief probabilities toward configurations of state variables that are discordant from the true state, decreasing the probability of the true state. Thus, the proposed algorithm adapts (i.e., changes) the MRF potentials when the agent acquires knowledge about the true state-variable values and detects a mismatch between the information in the learned MRF and the specific state-variable relationships of the episode to fix the mismatch. The adaptation is performed online, as POMCP works, limiting the performance decrease that could derive from the usage of the MRF when the true state-variable configuration represents an unlikely state.

Our empirical analysis shows that the MRF adaptation method improves the performance obtained using the MRF without adaptation. We tested the algorithm on two domains, namely, rocksample (Smith and Simmons, 2004), a benchmark domain in which an agent moving in a grid has to collect hidden rocks maximizing their values, and velocity regulation (Castellini et al., 2020, 2021), a domain in which a robot traveling on a predefined path has to regulate its velocity to minimize the time to reach the end and the collisions with obstacles in the path. Results show an average improvement of a discounted reward of 6.54% on rocksample and 3.51% on velocity regulation. Finally, we tested the proposed ROS-based architecture on a Gazebo simulator of rocksample. The architecture enables the generation of informative MRFs that produces statistically significant performance improvements. A video showing the evolution of the learning process performed on the ROS-based architecture and the Gazebo simulator is available in the Supplementary Material. The code of the ROS-based architecture is also available online.^1^

In summary, the main contributions of this work to state of the art are as follows:

• We present a methodology for learning state-variable relationships in the form of an MRF as POMCP is executed on a mobile robot.
• We introduce a framework to integrate POMCP within ROS, targeting ROS-based mobile robots. The architecture supports both the phase in which the MRF is learned and the phase in which it is used.
• We propose an algorithm for adapting the MRF constraints to episodes having unlikely state-variable configurations as new observations are acquired from the environment.

The rest of the study is organized as follows: Section 2 discusses related work. Section 3 describes the rocksample domain used as a running example. Section 4 presents background on POMDP, POMCP, MRF, and the extended POMCP. Section 5 formalizes the learning algorithm and the stopping criterion, describes the ROS-based architecture, and formalizes the MRF adaptation method. Section 6 presents the empirical evaluation of the three contributions. Section 7 draws conclusions and suggests future research directions.

## 2 Related Work

We identified four research topics in the literature related to our work: probabilistic planning under uncertainty, application of POMCP to robotic platforms, Bayesian adaptive learning and other forms of learning for planning, and MRF learning.

Planning under uncertainty is a crucial task for autonomous and intelligent agents. The first works on POMDP-based planning date back to the seventies (Sondik, 1978). Since then, several methods have been proposed to solve POMDPs (Kaelbling et al., 1998). Recent works highlight the benefits of introducing prior knowledge in problems formalized as POMDPs and solved by POMCP. Castellini et al. (2019) showed that the introduction of prior knowledge about state-variable relationships yields performance improvement. In particular, constraints expressed as MRFs (Murphy, 2012) and CNs (Dechter, 2003) were used. Castellini et al. (2021) showed how mobile robots exploited prior knowledge about task similarities to improve their navigation performance in an obstacle avoidance context. The main limitation of these works regards the requirement to have a full specification of the prior knowledge in advance, but this is not always feasible in practice, especially in complex application domains such as robotic ones. What differentiates our work from Castellini et al. (2021, 2019) is that here we aim to learn the MRF on real robots while acting in the environment and adapt the MRF while it is used. Some other works deal with the problem of adding constraints to planning for improving the performance or scaling to large environments. Lee et al. (2018)used MCTS to generate policies for constrained POMDPs, and Amato and Oliehoek (2015) explored the multi-agent structure of some specific problems to decompose the value function. Instead, we constrain the state space on the basis of state-variable relationships to refine the belief during execution. More precisely, we exploit the learned MRF whose potentials express probabilistic constraints between state-variable values. Other related works in the field of planning under uncertainty concern factored POMDPs and their applications (McAllester and Singh, 1999; Williams and Young, 2007). However, our approach is substantially different as the performance improvement does not derive from a factorization of the POMDP but from the introduction in POMDP of prior knowledge on the domain, represented as an MRF learned from previously collected data.

Regarding the application of POMCP to robotic platforms, we noticed that the planning algorithm has been recently applied to different robotic problems. Goldhoorn et al. (2014) proposed two extensions of POMCP to find-and-follow people that work in the continuous space and plan actions in real time. The Adaptive Highest Belief Continuous Real-Time POMCP Follower presented in that paper aimed to avoid unnecessary turns of the robot in reaching the goal. Our method and ROS-based architecture, instead, aim to learn state-variable relationships and use them in POMCP to improve planning performance. Wang et al. (2020) and Giuliari et al. (2021) used POMCP in the context of Active Visual Search. The authors proposed a method in which the agent starts acting in an unknown environment (i.e., with no information about the area map). Moreover, they present a new belief reinvigoration approach dealing with dynamically growing state space. Lauri and Ritala (2016) used POMCP to control a mobile robot to explore a partially known environment. POMCP was previously integrated with ROS in Wertheim et al. (2020), where a robotic planning platform called ROS-POMDP was presented. It generated the POMDP model of the problem using Performance Level Profiles (PLP) (Brafman et al., 2016) and Relational Dynamic Influence Diagram Language (RDDL) (Sanner, 2010). A two-layer control architecture was instead proposed by Castellini et al. (2021), where the upper layer used an extension of POMCP to tune the velocity of a mobile robot and the lower layer used a standard engine controller to deal with path planning. As explained above, our proposed ROS architecture has a completely different goal, integrating the learning of the MRF with POMCP.

As our goal is to learn some information about the environment and introduce it in POMCP to improve its performance, we also analyzed related works on merging learning and planning, with a specific focus on POMDPs and POMCP. Our work is also related, for instance, to Bayesian adaptive learning in POMDPs (Ross et al., 2011). Katt et al. (2017) presented an elegant method for learning the transition and reward models. They extended the POMCP algorithm to the Bayes-Adaptive case, proposing the Bayes-Adaptive Partially Observable Monte Carlo Planning (BA-POMCP) approach that, however, learns the parameters of the transition model. Our method, instead, learns probabilities of pairs of state variables to have equal values in the hidden part of single states (i.e., we do not consider any information about how the state changes over time). We assume that the hidden part of the state can change only from one episode to another, and each state has a probability of occurring that depends on some (unknown) state-variable probabilistic relationships. We notice that this setting is very common in practice (see the warehouse example in the introduction), but it cannot be naturally encoded in the transition model. The information encoded in our MRF is instead used to initialize and update the belief. For the same reason, our approach also differentiates from Factored BA-POMDP (Katt et al., 2019), which learns a compact model of the dynamics by exploiting the underlying structure of a POMDP, allowing for better scale to large problems. Even this approach deals with knowledge about the transition from one state to another across the steps of execution, and it cannot learn the probability distribution of states considering probabilistic state-variable relationships, as our MRF does. We remark that we do not factorize the POMDP to learn the compact model of dynamics. We are interested in learning probabilistic relationships between state-variable values, which is information affecting the belief and its update over time. For instance, the traffic level in two aisles of a warehouse can be highly correlated. Hence, in an episode, the two aisles may have a high traffic level; in another episode, they may have a low traffic level, but the probability that the two aisles have different traffic levels in an episode is low. This prior knowledge about the state of the environment, represented by the initial belief in POMDPs, can be naturally integrated into POMCP using the MRF, a generative model that directly represents state-variable relationships. Using the MRF, we push the belief probabilities toward states that agree with this knowledge. Methodologies for optimally updating POMDP beliefs to reduce uncertainty on the true state have been proposed by Stachniss et al. (2005), Araya et al. (2010), Veiga (2015), Ognibene et al. (2019), Fischer and Tas (2020), and Thomas et al. (2020). However, these methods mainly focus on introducing the belief into the reward function to allow the definition of information gain goals, otherwise not definable, in the context of POMDP. In order to deal with large environments in practical problems, hierarchical models (Friston, 2008) have been used to extend the POMDP framework (Pineau et al., 2001; Theocharous et al., 2001; Theocharous et al., 2004; Sridharan et al., 2008; Doshi-Velez, 2009). These approaches take advantage of the structure of the problem to decompose the state or the action space, introducing different levels of abstraction to learn much larger models. Moreover, in these works, the computation of optimal policies is performed considering only a subset of the models or an action subset because it is intractable to compute optimal policies for the original problem. However, in our approach, we do not decompose the original problem into sub-tasks. We compute policies considering the entire problem domain. Finally, within the research topic of learning for planning in robotic platforms, Atrash and Pineau (2010) proposed a methodology for learning a model of the user in applications where untrained humans interact and control the robot. In this case, the goal is also to learn a model of the environment.

In the literature, some works proposed approaches to learning arbitrary MRF structures (Besag, 1977; Abbeel et al., 2006; Pletscher et al., 2009; Salakhutdinov, 2009; Vuffray et al., 2020) mainly in the field of computer vision. Due to their generality, these approaches have a higher complexity than our proposed approach, which is specialized in pairwise MRF for representing state-variable relationships inside POMDPs. Shah et al. (2021) also focused on pairwise MRF, but their proposed methodology focused on learning continuous pairwise MRF. The MRFs that we used in our approach are discrete.

## 3 Rocksample: A Domain for a Running Example

As a running example for explaining the main elements of the proposed contributions, in the rest of the study, we consider rocksample (Smith and Simmons, 2004) a benchmark domain inspired by robotic planetary exploration. In the rocksample, the agent acts in a grid containing valuable and valueless rocks and aims to maximize the value of the collected rocks. The agent does not know the values of the rocks, but it knows only their locations in the grid. Rock values can only be inferred from noisy observations returning the true value of the rock with a probability proportional to the distance between the agent and the observed rock. Knowing in advance the relationships between pairs of rock values (e.g., close rocks could have similar values in real-world applications), the agent can improve its planning performance, collecting more valuable rocks in less time. An example of a probabilistic equality relationship between two state variables *X*_1_ and *X*_2_ assuming values in {0, 1} is “*X*_1_ is equal to *X*_2_ with probability 0.9”. In the rocksample, this means that rocks 1 and 2 have the same values with high probability. This kind of relationship cannot be encoded in the transition or observation models because it does not deal with the dynamics of the environment or state observability. Instead, it is a property of state distribution and can be represented by the potential of a pairwise MRF in which nodes correspond to state variables and edges to probabilistic relationships between pairs of state-variable values.

## 4 Background

In this section, we provide definitions of POMDP, the model used to formalize our planning problem, POMCP, the planning algorithm used to solve the POMDP, MRF, and the structure used to represent state-variable relationships. Finally, we describe the extension of POMCP that considers prior information.

### 4.1 POMDP

A POMDP (Kaelbling et al., 1998) is defined as a tuple (*S*, *A*, *O*, *T*, Ω, *R*, *γ*), where *S* is a finite set of *states*, *A* is a finite set of *actions*, Ω is a finite set of *observations*, *T*: *S* × *A* → Π(*S*) is the *transition* model, where Π(*S*) is the space of probability distribution over states, *O*: *S* × *A* → Π(Ω) is the *observation model*, R:S×A→R is the *reward function*, and *γ* ∈ [0, 1) is the *discount factor*. The agent’s goal, as in a MDP (Russell and Norvig, 2010), is to maximize the *expected discounted return* $E\left[\sum_{t=0}^{\infty }\gamma^{t}R\left(s_{t},a_{t}\right)\right]$ acting optimally (i.e., choosing, in each state *s*_*t*_, at time *t*, the action *a*_*t*_ with the highest expected reward). In the POMDP framework, however, the agent cannot directly observe the current state *s*_*t*_, but it maintains a probability distribution over states *S*, called *belief* which updates at each time step. In the following, we represent by symbol *b*(*s*) the probability of being in state *s* according to belief *b*. The belief summarizes the agent’s previous experiences, that is, the sequence of actions and observations that the agent took from an initial belief *b*_0_ to the belief *b*. The sequence of actions and observations is called history (*h*) and is represented as *h* = ⟨*a*_0_, *o*_0_, … , *a*_*t*_, *o*_*t*_⟩. The solution of a POMDP is an optimal or approximated *policy*, namely, a function that maps belief states into actions, that is, *π*: *B* → *A*, where *B* is the belief space. A policy is optimal if it maximizes the expected discounted return. The discount factor *γ* guarantees convergence by reducing the weight of long-term rewards.

### 4.2 POMCP

POMCP (Silver and Veness, 2010) is a Monte-Carlo-based algorithm for planning in partially observable environments that combines MCTS (Browne et al., 2012) to compute an approximated policy with a *particle filter* to represent the belief. The particle filter is initialized with *k* particles, each representing a state *s* and following a uniform distribution if no prior knowledge is available about the initial state. At each step, POMCP uses an MCTS to find the best action to perform. The MCTS is generated by iteratively 1) sampling a state from the particle filter and 2) performing a simulation with that state according to the transition and observation models known by the agent. The Upper Confidence bounds applied to the Trees (UCT) strategy (Kocsis and Szepesvári, 2006) is used to balance exploration and exploitation in the simulation phase. The reward of each simulation is backpropagated in the tree to compute the approximated Q-values *Q* (*b*, *a*) for the current belief *b* and, at the end of the process, the action *a* with the higher Q-value is selected. After the selected action, *a* is performed in the real environment, a real observation *o* is collected, and particles in the belief are updated by keeping only particles that explain the observations. Particle reinvigoration is used if no more particles are available in the particle filter.

### 4.3 Markov Random Fields

An MRF is an undirected graph where nodes represent variables and edges represent probabilistic relationships between variable values (Bishop, 2006; Murphy, 2012). A potential function is a non-negative function of its arguments representing the relative “compatibility” of different variable assignments. According to the Hammersley–Clifford theorem (Upton and Cook, 2008), the joint probability represented by the MRF can be computed as the product of potential functions over the maximal cliques of the graph, namely,

$$p\left(x|\theta \right)=\frac{1}{Z\left(\theta \right)}\underset{c\in C}{\prod }\psi_{c}\left(x_{c}|\theta_{c}\right),$$ (1)

where ***x*** is a variable configuration (e.g., ***x*** = (1, 0 … , 0)), ***θ*** is a parametrization of the MRF (i.e., a specific set of values for the parameters *θ* that represent the MRF), *C* is the set of maximal cliques, *ψ*_*c*_ (***x***_*c*_| ***θ***_*c*_) is the potential function, and *Z*(***θ***) is the partition function, that is, a normalization factor that can be computed as

$$Z\left(\theta \right)=\underset{x}{\sum }\underset{c\in C}{\prod }\psi_{c}\left(x_{c}|\theta c\right).$$ (2)

Potentials can be represented by a Boltzmann distribution (i.e., exponentials); thus, *ψ*_*c*_ (***y***_*c*_| ***θ***_*c*_) = exp (−*F* (***x***_*c*_| ***θ***_*c*_)), where *F* (***x***_*c*_) is the energy function. Restricting the parametrization of the MRF to the edge rather than to the maximal clique of the graph, we obtain *pairwise MRF*, and, consequently, the product of potentials can be computed by summing the energies of all pairwise relationships. We call *E* the set of pairwise relationships (*i*, *j*) in the MRF, where *i*, *j* ∈ 1, *…* , *n*, and *n* is the number of state variables. For instance, given a pair of state variables (*X*_*i*_, *X*_*j*_)|(*i*, *j*) ∈ *E* representing two rocks in rocksample, a potential could be $\psi_{X_{i},X_{j}}\left(0,0\right)=0.45$ , which indicates a compatibility of 0.45 to have value 0 in both rocks *X*_*i*_ and *X*_*j*_, or $\psi_{X_{i},X_{j}}\left(0,1\right)=0.05$ , which indicates a compatibility of 0.05 to have value 0 in rock *X*_*i*_ and 1 in rock *X*_*j*_. In the following, when we refer to an MRF we mean a set of potentials representing compatibilities of different variable assignments:

$$\psi_{X_{i},X_{j}}\left(l,h\right),\;\left(i,j\right)\in E,\;l,h\in \left\{1,\ldots ,k\right\},$$ (3)

where *k* is the number of possible values of each variable.

### 4.4 Extended POMCP

The methodology we use to introduce prior knowledge in POMCP (Castellini et al., 2019) allows for defining probabilistic equality relationships among pairs of state variables through MRFs. The use of the MRF allows factorizing the joint probability function of state-variable configurations, and this probability is used to constrain the state space. Indeed, the MRF defines a probability distribution over states of the POMDP. For instance, in the rocksample domain, the state space is the set of all possible rock value configurations, and the constraints introduced by the MRF allow (probabilistically) reducing the possibility of exploring states that have a small probability of being the true state. The integration of prior knowledge in POMCP is mainly developed in the particle filter initialization and in the reinvigoration phase (Castellini et al., 2019), where the probabilistic constraints stored in the MRF are used to optimize the management of the particle filter representing the agent belief.

**FIGURE 1:**
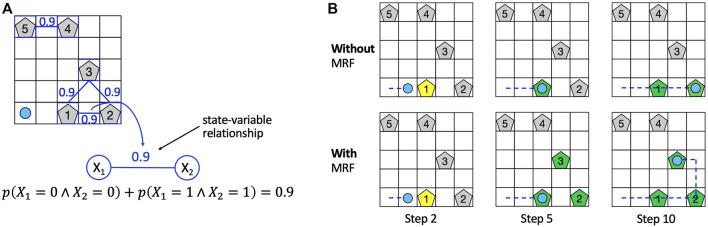
**(A)** Example of usage of the MRF in the rocksample environment. The MRF topology is depicted using rocks as nodes, and equality probability constraints are specified on blue edges between rocks. **(B)** Effect of the action performed by the agent at steps 2, 5, and 10 using standard POMCP (first row) and the information stored in the MRF (second row). A yellow-colored rock means that the rock is checked by the agent, whereas a green-colored rock represents a rock observed to be valuable by the agent. We consider rocks 1, 2, and 3 valuable in this example.

To intuitively understand the advantage introduced by the MRF, consider the rocksample environment depicted in Figure 1A, in which the knowledge introduced by the MRF is represented by blue edges between rocks on the grid. The prior knowledge about state-variable relationships is information about equality relationships among the value of different rocks (e.g., with a probability of 0.9, rocks 4 and 5 have the same value). We use this knowledge to “push” the belief probabilities toward states that agree with this information during particle filter initialization. The rationale is that if the agent knows that two rocks (i.e., two state variables) have the same value with high probability (0.9 in Figure 1A), then it can improve its planning performance because once it has observed the value of one rock, it has also acquired some knowledge about the value of the other rock and it can plan accordingly. In the first row of Figure 1B, we show a hypothetical sequence of action performed by the agent with no knowledge about rock values relationships (i.e., standard POMCP), whereas in the second row, we show a hypothetical sequence of action performed by exploiting such knowledge. In both cases, in step 2, the agent performs a sensing action to check the value of rock 1 (yellow colored). In the hypothesis that the agent observes that rock 1 is valuable (green pentagon in the second column), in the first case (i.e., without MRF), it has no information about rocks 2 and 3, whereas in the second case (i.e., with MRF), the agent also has some information about rocks 2 and 3, which are considered valuable with high probability (green pentagons). In step 10, exploiting the acquired knowledge about rock value relationships, the agent with MRF has already sampled rocks from the three rocks, whereas the agent without any knowledge has only sampled rocks 1 and 2. Hence, the agent with the MRF moves faster. We remark that the knowledge in the MRF does not affect the transition model but only the probability distribution over POMDP states. The knowledge stored in an MRF is used to initialize the particle filter (representing the belief) of POMCP and update the particle filter (i.e., the belief) during reinvigoration, a procedure used by POMCP to introduce new particles upon depletion.

## 5 Methodology

In this section, we present a method for learning the MRF during POMCP execution (Section 5.1) that leverages information from the state with the highest probability in the belief and a stopping criterion based on the convergence of MRF potentials (Section 5.2). In Section 5.4, we describe *MRF Adaptation*; the algorithm that adapts the learned MRF as new knowledge is gathered about the true state-variable configuration and it differs from the information in the MRF. Finally, in Section 5.3, we present the ROS-based architecture designed to have POMCP running within ROS and learn the MRF on real mobile robots.

### 5.1 MRF Learning

We present a method to learn the MRF during POMCP execution based on the information provided by the belief. More precisely, it employs information from the state having maximum probability. In all our tests, we assume to learn the MRF in *NE* episodes, where each episode *e* is composed of a fixed number of steps, but the proposed methodology can simply adapt to the case of episodes with a different number of steps. We assume the hidden part of the state to be static in each episode and changing across episodes. We initialize the MRF with uninformative priors and then update it at the end of each episode. Then, at the end of the learning process, we have an MRF which defines probabilistic constraints on hidden state variables. This information allows for better initializing and updating of the state distribution. Details about the proposed methodology and the used data structures are reported in the following.

#### 5.1.1 Data Structures Used in the Learning Algorithm

Learning the MRF means learning the potentials of pairwise MRF representing state-variable relationships. Given two variables *X*_*i*_ and *X*_*j*_ with *k* possible values each, we need to learn the potential $\psi_{X_{i},X_{j}}\left(l,h\right)$ for each pair (*l*, *h*) with *l* ∈ {1, *…* , *k*} and *h* ∈ {1, *…* , *k*}, where variable equality occurs when *l* = *h* and variable inequality occurs in all other cases. To keep track of state-variable values in different episodes, we use three data structures. First, a *vector of state-variable values* $V_{e}\left(i\right)$ for each episode *e*. $V_{e}\left(i\right)\in \left\{1,\ldots ,k\right\}$ is the value of the state-variable *X*_*i*_ extracted from the state with maximum likelihood in the final belief of episode *e* (*i* = 1, *…* , *n* where *n* is the number of state variables). This vector is initialized to $V_{e}\left(i\right)=0,\forall i\in \left\{1,\ldots n\right\}$ , and then each value $V_{e}\left(i\right)$ is updated to the value in {1, … , *k*} obtained for variable *X*_*i*_ in episode *e*. The second data structure is a four-dimensional array in which we store the *count of equalities and inequalities* among pairs of state variables in each episode *e*, $M_{e}\left(i,j,l,h\right)$, where (*i*, *j*) ∈ *E* and *l*, *h*, ∈ {1, *…* , *k*}. The value $M_{e}\left(i,j,l,h\right)$ is the number of times variable; *X*_*i*_ had value *l* and variable *X*_*j*_ had value *h* in the previous *e* episodes, where e∈N . We update $M_{e}$ at the end of each episode *e* using the values in $V_{e}\left(i\right)$ , and the MRF potentials $\psi_{X_{i},X_{j}}\left(l,h\right)$ are directly computed using values in $M_{e}\left(i,j,l,h\right)$ (Eq. 5). Hence, the MRF can be updated using values in $M_{e}$ . The third data structure is a matrix of *probabilities of state-variable equalities*, $P_{e}\left(i,j\right)$ , where (*i*, *j*) ∈ *E*. The value $P_{e}\left(i,j\right)$ is the probability that state variables *X*_*i*_ and *X*_*j*_ had equal values until episode *e* (Eq. 6). Notice that the proposed learning algorithm learns both equalities and inequalities’ probabilistic relationships. Equalities are represented by edges with positive probabilities (e.g., $P_{e}\left(i,j\right)=0.9$ means that rocks *X*_*i*_ and *X*_*j*_ have a 0.9 probability to have equal values), whereas inequalities are represented by edges with negative probabilities (e.g., $P_{e}\left(i,j\right)=0.1$ means that rocks *X*_*i*_ and *X*_*j*_ have only 0.1 probability to have equal values, viz, they have a probability 0.9 with different values).

In summary, at each episode *e*, we compute $M_{e}$ from $V_{e}$ , *ψ* from $M_{e}$ , and finally $P_{e}$ from *ψ* following the pipeline $V_{e}\left(i\right),V_{e}\left(j\right)\to M_{e}\left(i,j,l,h\right)\to \psi_{X_{i},X_{j}}\left(l,h\right)\to P_{e}$ . In the next section, we present the proposed learning algorithm and the related strategy for populating $V_{e}$ and update $M_{e}$ .

#### 5.1.2 Learning Algorithm

At each episode *e*, the vector of state-variable values $V_{e}\left(i\right)$ is first populated with the values of state variables *X*_*i*_ of the state having maximum likelihood in the agent belief.

**Update of equality/inequality counts M .** The array of equality/inequality counts M is initialized to M(i,j,l,h)=0 , *∀*(*i*, *j*) ∈ *E*, *∀l*, *h* ∈ {1, … *k*}. At the end of each episode, the array $M_{e}$ is updated using vector $V_{e}$ as

$$M_{e+1}\left(i,j,l,h\right)=\left\{\begin{array}{cc} M_{e}\left(i,j,l,h\right)+1\; & \text{ if }V_{e}\left(i\right)=l\wedge V_{e}\left(j\right)=h\\ M_{e}\left(i,j,l,h\right)\; & \text{ otherwise} \end{array}\right..$$ (4)

**Computation of potentials *ψ* from counts M .** We compute MRF potentials *ψ* from multi-dimensional array M at each episode *e* by normalizing each cell using the following formula:

$$\psi_{X_{i},X_{j}}^{e}\left(l,h\right)=\frac{M_{e}\left(i,j,l,h\right)}{\sum_{w=1}^{k}\sum_{y=1}^{k}M_{e}\left(i,j,w,y\right)},$$ (5)

where (*i*, *j*) ∈ *E*. Namely, we consider only pairs of nodes connected by an edge. For instance, given a pair of state variables (*X*_*i*_, *X*_*j*_)|(*i*, *j*) ∈ *E* assuming values in {0, 1}, the potential $\psi_{X_{i},X_{j}}^{e}\left(0,0\right)=0.6$ corresponds to the ratio between the number of times *X*_*i*_ = *X*_*j*_ = 0 and the number of times each possible assignment for *X*_*i*_ and *X*_*j*_ has been observed. Namely, given $M_{e}\left(i,j,0,0\right)=6$ , $M_{e}\left(i,j,0,1\right)=1$ , $M_{e}\left(i,j,1,0\right)=1$ , and $M_{e}\left(i,j,1,1\right)=2$ , we compute $\psi_{X_{i},X_{j}}^{e}\left(0,0\right)=\frac{6}{6+1+1+2}=0.6$ .

**Computation of probabilities of state-variable equalities P from *ψ*.** These probabilities are finally computed for each (*i*, *j*) ∈ *E*:

$$P_{e}\left(i,j\right)=\overset{k}{\underset{l=1}{\sum }}\psi_{X_{i},X_{j}}^{e}\left(l,l\right).$$ (6)

In other words, $P_{e}\left(i,j\right)$ is the sum of potentials corresponding to equal values of variables *X*_*i*_ and *X*_*j*_. For instance, given the pair of state variables (*X*_*i*_, *X*_*j*_) and the potentials $\psi_{X_{i},X_{j}}^{e}\left(0,0\right)=0.6$ , $\psi_{X_{i},X_{j}}^{e}\left(0,1\right)=0.1$ , $\psi_{X_{i},X_{j}}^{e}\left(1,0\right)=0.1$ , and $\psi_{X_{i},X_{j}}^{e}\left(1,1\right)=0.2$ , we compute $P_{e}\left(i,j\right)=0.8$ .

### 5.2 Stopping Criterion

At the end of each learning episode, the MRF is updated, considering the information about the state-variable relationships acquired in the episode. The question we answer in this section is, “when can the learning process be stopped?”. The MRF must provide meaningful knowledge about state-variable relationships to improve planning performance. The methodology we propose analyzes the equality probabilities in the MRF and stops the learning phase when these probabilities converge, namely, when their values have little changes for a few consecutive episodes. More precisely, at the end of each episode *e*, we check if each equality probability $P_{e}\left(i,j\right),\;\left(i,j\right)\in E$ , differs less than a threshold *η* from the same equality probability at the end of the previous episode *e* − 1. If this condition is satisfied for *ce* consecutive episodes, then we stop the MRF learning process. Algorithm 1 formalizes the approach. It receives the matrices of equality probabilities at episodes *e* and *e* − 1, namely, $P_{e}\left(i,j\right)$ and $P_{e-1}\left(i,j\right)$ , the convergence threshold *η*, the threshold *ce* on the number of consecutive episodes, and the number *ct* of consecutive episodes that satisfied the condition on the convergence threshold until the current episode *e*. It returns the stop learning flag *stop* and the updated number of consecutive episodes that satisfy the convergence condition $\bar{ct}$ . The value of *stop* is true if, for every edge, the difference between the value at episode *e* and episode *e* − 1 is below the threshold *η* (line 3) for at least three consecutive episodes (line 9), false otherwise. The value of $\bar{ct}$ is used for checking the stopping condition at the next episode.

Algorithm 1 Stopping criterion.

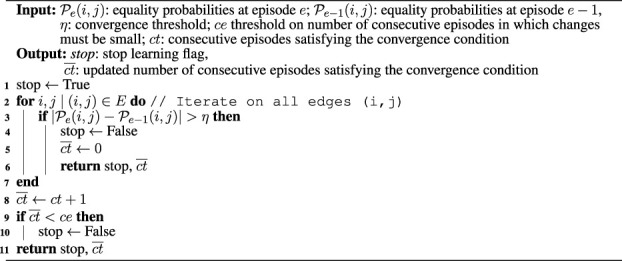

### 5.3 ROS Architecture for POMCP

**FIGURE 2:**
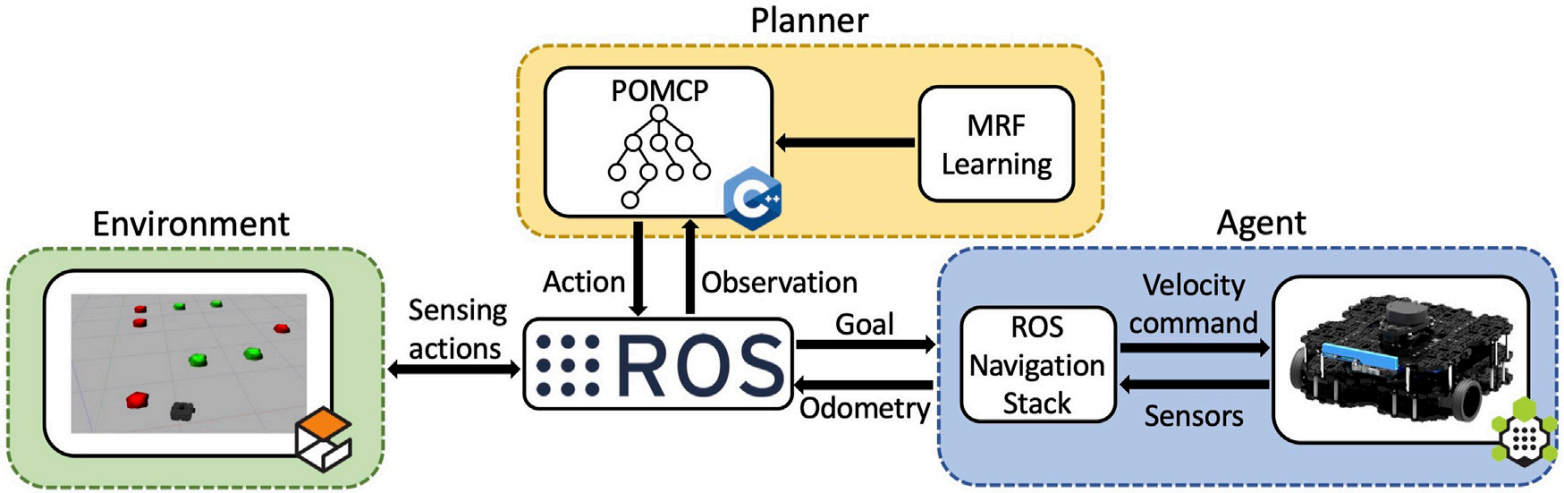
ROS architecture for running POMCP on mobile robotic platforms. The three main components are identified by the colored boxes and connected to the same ROS network. The planner supports standard POMCP, extended POMCP, and our proposed approach with MRF adaptation, besides the MRF learning algorithm.

We developed a light and straightforward framework that integrates POMCP with ROS, targeting mobile robots. The architecture can also be exploited to execute the MRF learning algorithm and subsequently run the extended POMCP that leverages the constraints in the learned MRF. Optionally, the extended POMCP can be run with MRF adaptation. The architecture can be used with all mobile robotic platforms supporting the ROS Navigation Stack (Marder-Eppstein et al., 2010) and with POMDPs defined following the original POMCP implementation. Additionally, it can be executed in simulation using Gazebo. Since the architecture relies on the ROS network to communicate, the POMCP algorithm is not directly run on the machine mounted on the robotic platform but on an external one, which has more computational power. This results in faster execution of POMCP, with lower power consumption for the mobile robot. The structure of the architecture is illustrated in Figure 2. It contains three main components, namely, the *environment*, the *planner*, and the *agent*, all implemented in C++. In the following paragraphs, each component is described in detail.

#### 5.3.1 Environment

The environment is a discretization of the real world that exploits a task-specific representation, such as a grid for the rocksample domain.

#### 5.3.2 Planner

The role of the planner is manifold. First, it runs POMCP, from the standard to the extended version. Second, it manages the whole learning process, handling the learning algorithm and keeping track of learned relations to eventually trigger the stopping criterion. When performing the MRF learning process, the node keeps track of the belief, after which each action is performed by the agent. Then, at the end of each episode, the MRF is updated accordingly.

The planner communicates with the ROS network during the *Step* function call, hence when applying the transition and the observation model of the POMPD. Right after producing the best-desired action, a command is dispatched to the agent, and the planner pauses until the agent feeds back the result.

#### 5.3.3 Agent

The agent node is the interface with the robotic platform. It holds information about the robot’s position through odometry and is responsible for moving the mobile platform to the desired position whenever the planner produces a goal command, which corresponds to a 3D pose in the environment. This is done by exploiting the ROS Navigation Stack, which takes the pose as input and gives a series of target velocities as output. On the contrary, if the planner produces a sensing action, the agent will directly interfere with the environment or sensors mounted on the robotic platform.

### 5.4 MRF Adaptation

The MRF is learned on several episodes and contains probabilistic information about state-variable relationships. For instance, a probability of 0.9 between state variables *X*_1_ and *X*_2_, that is, P(1,2)=0.9 , in the rocksample domain means that, in 90% of the learning episodes, the most probable state-variable configuration had equal values in rocks *X*_1_ and *X*_2_. When the MRF is used in a new episode, however, the values of the rocks in that specific episode can be equal to or different from each other (e.g., in a specific episode *X*_1_ could be valuable and *X*_2_ valueless, although this configuration has only probability 0.1 to occur). The MRF is used in POMCP to “push” the belief probabilities toward states that agree with the joint probability it represents. In other words, using the constraints among state-variable values introduced by the MRF, we probabilistically reduce the possibility of having in the particle filter a large number of particles corresponding to states with a small probability of being the true state. At each episode, we initialize the belief leveraging the information present in the MRF, peaking the probability distribution on states that reflect the equality relationships expressed in the MRF. In our example, the states with the same value of *X*_1_ and *X*_2_ will be initialized with a higher probability. This is beneficial if the values in the true state of the current episode are actually equal (which happens with a probability of 0.9 in our example) and harmful if the values in the true state of the current episode are actually different from each other (which happens with a probability of 0.1). In this second case, the belief is peaked in the wrong states and even several observations could be not enough to “correct” the probability distribution over states, leading to a performance decrease with respect to the standard POMCP. Thus, the idea of the algorithm presented in this section is to adapt the probabilities in the MRF during the usage of the MRF as new evidence is gathered about the true values of the state variables in the specific episode and there is a mismatch (i.e., *discrepancy*) between these true values and the information in the MRF. Then, the adapted MRF is used to re-initialize the belief to change the agent strategy. Let us consider, for instance, an episode of rocksample in which rock *X*_1_ is valuable and rock *X*_2_ is valueless. When we use the MRF, the states with different rock values are penalized, but if the agent collects the rocks, then their true values are available. Hence, we can detect the discrepancy between the MRF probabilities P(1,2)=0.9 and the true rock values and update the MRF probabilities accordingly to avoid penalizing good states in the following steps of the same episode. This is the idea behind the MRF adaptation algorithm formalized in Algorithm 2 and explained in detail in the following: notice that, given an episode *e*, the adaptation of the MRF has effect only in the steps after a discrepancy is detected in that episode. However, the learned MRF is restored in the next episode *e* + 1 because each episode is characterized by a different true state. We remark that the proposed algorithm does not learn a new MRF, as it adapts the information stored in the learned MRF when a discrepancy is detected during an episode, and it uses the adapted MRF to re-initialize the particle filter. At the beginning of the subsequent episode, we restore the learned MRF (with no adaptation) and use it to initialize the particle filter. On the contrary, during the learning process, we update the MRF leveraging the information given by the state with the highest probability in the belief, and we do not introduce the MRF in POMCP.

Algorithm 2 MRF adaptation algorithm.

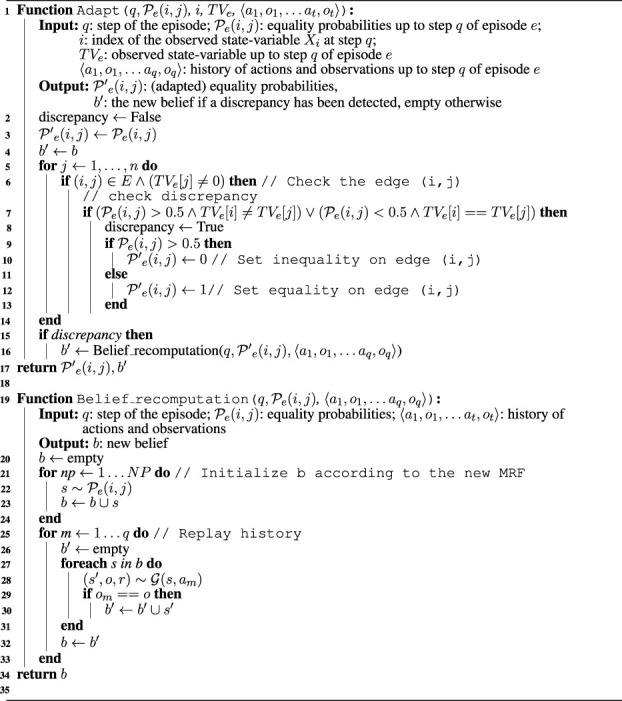

The inputs of the main function of the algorithm (Function Adapt) are as follows: the step *q* of the episode, the MRF P(i,j),i=1,…,n,j=1,…,n updated until the current step of the current episode; the index *i* of the state-variable of which we have observed the true value in the current step; the vector *TV* [*i*] of state variables observed until the current step, where *TV* [*i*] = *na* if the true value of the variable has not been observed and *TV* [*i*] = *v*_*i*_ if the true value of the variable has been observed; and ⟨*a*_0_, *o*_0_, … , *a*_*q*_, *o*_*q*_⟩ the sequence of actions and observations (history) obtained up to the current execution step *q*. The output is the adapted MRF and the new belief *b*′ (returned by Function Belief_recomputation) if a discrepancy has been detected. Otherwise, the Function Adapt ends returning the received MRF and an empty belief to notify that no discrepancies have been detected.

Every time the true value of a state-variable *X*_*i*_ is gathered, the algorithm checks if *X*_*i*_ is connected to another state variable *X*_*j*_ by an edge in the MRF and if both variables have been observed (line 6). In this case, the algorithm checks the value of P(i,j) and the values of the observed variables *v*_*i*_ and *v*_*j*_ to detect a discrepancy (line 7). In particular, a discrepancy occurs if the equality probability in the MRF is discordant with the true values of *X*_*i*_ and *X*_*j*_. In such a case, the MRF must be updated. If P(i,j)>0.5 and *v*_*i*_ ≠ *v*_*j*_, then P(i,j) is set to 0 (see line 10). This is because we are sure that the two variables have different values in the current episode, and the MRF is updated accordingly. If P(i,j)<0.5 and *v*_*i*_ = *v*_*j*_, then the algorithm sets P(i,j) to 1 (see line 12). In this case, we are sure that the two variables have the same values, and the MRF is again updated accordingly. In the example above, if rock *x*_1_ is valuable and rock *X*_2_ is valueless but P(1,2)=0.9 , then this probability is set to P(1,2)=0 in the adapted MRF.

If a discrepancy has been detected and the MRF updated, the current belief at step *q* must also be updated considering the new specific knowledge acquired on the current episode. Function *Belief_recomputation* performs this task. Its inputs are the step *q* of the episode, the adapted MRF P(i,j) , and the history of actions and observations ⟨*a*_0_, *o*_0_, … , *a*_*q*_, *o*_*q*_⟩, and its output is the updated belief *b*. The new belief *b* is first initialized (line 21), sampling *NP* states according to the distribution defined by the adapted MRF (we set *NP* to the number of POMCP simulations), and then updated using POMCP belief update following the current history ⟨*a*_1_, *o*_1_, … *a*_*q*_, *o*_*q*_⟩ (see line 25). Silver and Veness (2010) used a simulator G as a generative model of the POMDP. The updated belief *b* is used in the next step instead of the current belief.

## 6 Experiments

In this section, we present the results of our empirical analysis. We perform three different tests on two application domains described in Section 6.1. Section 6.2 defines the measure used to evaluate the performance. Then, we present the results of our test following the order by which we introduced the methodological contributions. First, in Section 6.3, we analyze the performance of the proposed learning algorithm on a *C++* simulator of the rocksample environment. The empirical analysis shows the average performance improvement achieved using the learned MRF in the extended POMCP against standard POMCP (in the following, we refer to them as *EXT* and *STD*, respectively). Second, in Section 6.4, we show the evaluation of the ROS-based architecture for learning and using the learned MRF. The empirical analysis shows the average performance improvement achieved when the MRF learned on the robotic platform is used in EXT on the same platform. A video is also presented, which shows a complete learning process performed on the Gazebo simulator of rocksample. Third, in Section 6.5, we describe the experiments performed to evaluate the MRF adaptation algorithm. The performance of POMCP with MRF adaptation (*ADA*, in the following) is compared with that of EXT.

### 6.1 Domains

We provide full details on the two application domains used in our tests, namely, rocksample (Smith and Simmons, 2004) and velocity regulation (Castellini et al., 2020, 2021).

#### 6.1.1 Rocksample

**FIGURE 3:**
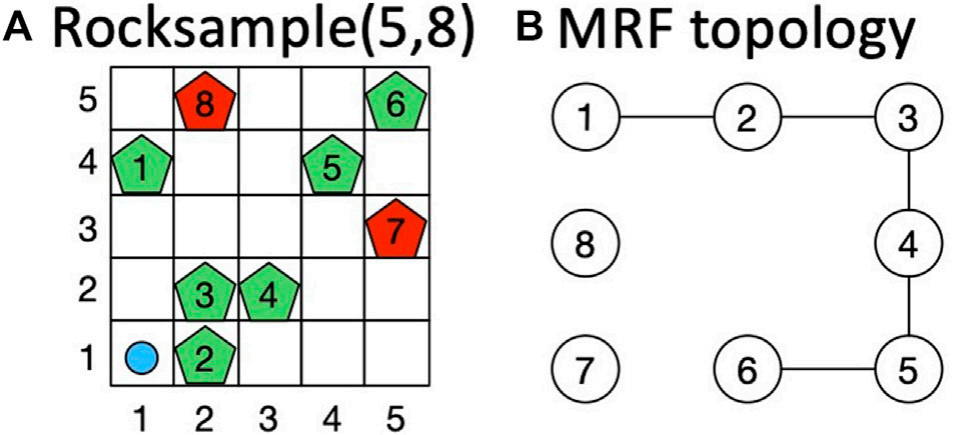
**(A)** Instance of rocksample environment (Zuccotto et al., 2022). **(B)** True MRF topology used for the domain.

In the rocksample domain (Smith and Simmons, 2004), an agent moves through a grid containing valuable and valueless rocks placed in a fixed position to maximize the discounted reward collecting rock values. We perform our tests on rocksample (5,8), consisting of a 5 × 5 grid in which we pose eight rocks (Figure 3A). The rock value configuration changes at each episode and is decided *a priori* to reflect specific constraints. Notation (*i*, *j*) identifies the cell in column *i* and row *j* on the grid, whereas for rocks, we use indices from 1 to 8. The agent (light blue circle in Figure 3A) knows the rock locations, but it cannot observe rock values (which is the hidden part of the state). These values can only be inferred using observations returned by the environment. The correct result of rock observations, however, is inversely proportional to the distance between the agent position and the rock. At each step, the agent performs one action among *moving* (up, down, left, right), *sensing* a rock (i.e., checking its value), or *sampling* a rock (i.e., collecting its value). The reward obtained by moving and sensing is 0, whereas sampling a rock gives a reward of 10 if the rock is valuable and −10 if it is valueless. Figure 3B shows the true MRF we used to constrain rock values. It presents five edges with the following probability values: P(1,2)=0.90 , P(2,3)=0.91 , P(3,4)=0.92 , P(4,5)=0.91 , and P(5,6)=0.91 . Thus, admissible configurations of rock values have, with high probability, all these rocks with the same value, whereas the values of rocks 7 and 8 can be randomly assigned because there are no constraints on their values in the MRF.

This problem can be formalized as a POMDP. The *state* is characterized by 1) the agent position on the grid, 2) the rocks’ configuration (hidden), and 3) a flag indicating rocks already sampled. The set of *actions* is composed of the four moving actions, the sample action, and a sensing action for each rock. *Observations* have three possible values: 1 for valuable and 2 for valueless rock observation returned by sensing actions and 3 for null observations returned by moving actions. The discount factor used is *γ* = 0.95. We aim to maximize the information learned about state-variable relationships, so we prevent the agent from exiting the grid.

#### 6.1.2 Velocity Regulation

**FIGURE 4:**
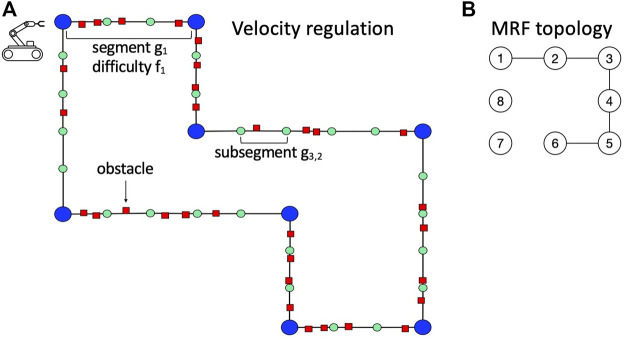
**(A)** Instance of velocity regulation. **(B)** True MRF topology used for the domain.

In the velocity regulation problem (Castellini et al., 2020, 2021), a mobile robot traverses a pre-defined path (Figure 4A) divided into segments *g*_*i*_ and subsegments *g*_*i*,*j*_. Notation (*i*, *j*) identifies the position of the robot in the path, where *i* is the index of the segment and *j* the index of the subsegment. More precisely, with (*i*, *j*), we mean that the agent is at the beginning of subsegment *g*_*i*,*j*_. Each segment is characterized by a difficulty *f*_*i*_ that depends on the obstacle density in the segment. The robot has to traverse the entire path in the shortest possible time, tuning its speed *v* to avoid collisions with obstacles. Each time the robot collides, a time penalty is given. The robot does not know in advance the real difficulty of the segments (which is the hidden part of the state), and it can only infer their values from the readings of a sensor (Figure 4A). Figure 4B shows the true MRF that we used to constrain segment difficulties. It presents five edges with the following probability values: P(1,2)=0.90 , P(2,3)=0.91 , P(3,4)=0.92 , P(4,5)=0.91 , and P(5,6)=0.91 . Thus, admissible configurations of segment difficulties have, with high probability, all these segments with the same value, whereas the values of segments 7 and 8 can be randomly assigned as there are no constraints on their values in the MRF.

This problem can be formalized as a POMDP. The *state* is characterized by 1) the position of the robot in the path; 2) the (hidden) true configuration of segment difficulties (*f*_1_, *…* , *f*_*m*_), where *f*_*j*_ ∈ {*L*, *M*, *H*}, *L* represents low difficulty, *M* medium difficulty, *H* high difficulty; 3) *t* is the time elapsed from the beginning of the path. The set of *actions* is composed of the three possible speed values of the robot in a subsegment: slow (*S*), intermediate (*I*), or fast (*F*). *Observations* are related to subsegment occupancy and robot angular velocity. The *occupancy model* *p* (*oc*|*f*) probabilistically relates segment difficulties to subsegment occupancy. *oc* = 0 means that no obstacles are detected in the next subsegment. On the contrary, *oc* = 1 means that some obstacles are detected. The *angular velocity model*, instead, provides the probability of angular velocity given segment difficulties, namely, *p* (*av*|*f*). More precisely, *av* = 0 means that the robot performs a few curves in the subsegment, whereas *av* = 1 means it performs several curves. In a realistic application on a mobile robot, *oc* is computed by averaging the values of the laser in front of the robot and applying a threshold to obtain the two binary values. Moreover, we count the actions corresponding to robot turns with angular velocity ≥45 °/s, and threshold such count to obtain the binary signal for *av*. The final observation is a coding of both variables *oc* and *av* computed as *o* = *av* + 2 ⋅ *oc*. Namely, *o* = 0 if *av* = 0 and *oc* = 0; *o* = 1 if *av* = 1 and *oc* = 0; *o* = 2 if *av* = 0 and *oc* = 1; and *o* = 3 if *av* = 1 and *oc* = 1. The observation model provides the probability of observations given segment difficulties, namely, *p* (*o*|*f*). We refer to the original work on the velocity regulation problem for more details about specific parameters (Castellini et al., 2021).

The time required to traverse a subsegment depends on the action that the agent performs and the time penalty it receives. Namely, the agent needs one time unit if the action is *F* (fast speed), two time units if the action is *I*, and three time units if the action is *S*. The *collision model* *p* (*c*|*f*, *a*) regulates the collision probability; more precisely, *c* = 0 means no collision and *c* = 1 means a collision occurs. The reward function here is *R* = −(*t*_1_ + *t*_2_), where *t*_1_ is the time depending on the agent’s action and *t*_2_ is the penalty due to collisions (in our tests *t*_2_ = 10). Finally, the discount factor we used is *γ* = 0.95. The parameters used in our tests are summarized in Tables 1–3.

**TABLE 1:** Main elements of the POMDP model for the collision avoidance problem. Occupancy model *p* (*o*|*f*): probability of subsegment occupancy given segment difficulty.

*f*	*p* (*oc* = 1 | *f*)
L	0.600
M	0.690
H	0.940

**TABLE 2:** Main elements of the POMDP model for the collision avoidance problem. Angular velocity model *p* (*av*|*f*).

*f*	*p* (*av* = 1 | *f*)
L	0.170
M	0.240
H	0.530

**TABLE 3:** Main elements of the POMDP model for the collision avoidance problem. Collision model *p* (*c*|*f*, *a*): collision probability given segment difficulty and action.

*f*	*a*	*p* (*c* = 1 | *f*, *a*)
L	S	0.000
L	I	0.033
L	F	0.033
M	S	0.000
M	I	0.033
M	F	0.067
H	S	0.000
H	I	0.067
H	F	0.100

### 6.2 Performance Measure

We introduce the performance measure used to evaluate the planning performance of our methods: difference and average difference in discounted returns. The discounted return of episode *e*, called *ρ*_*e*_, is the sum of the discounted rewards collected in all steps of that episode. The difference between the discounted return obtained using two different methods, such as EXT with the learned MRF and STD or ADA and EXT, on episode *e* is called Δ*ρ*_*e*_. The average of this difference over all episodes of all runs is called $\bar{\Delta \rho_{e}}$ . Notice that the difference is computed episode by episode to reduce the randomness, and the average is computed across all the episodes of each run. Indeed, the discounted return depends on the state of the episode; then, it could have very different values over the episodes and the distribution of these data would be very large. By computing the mean of the difference on each episode, we always compare the performance of the two algorithms in the same state, thus obtaining a low standard deviation value as a result of the reduced level of uncertainty.

### 6.3 Test on MRF Learning

We introduce the experimental setting used in our tests on the MRF learning method and then present our empirical analysis results.

#### 6.3.1 Experimental Setting

We perform tests using the MRF learning algorithm (Section 5.1) with the stopping criterion (Section 5.2) to learn the MRF. Experiments are performed on the rocksample domain described in Section 6.1.1 using a *C++* simulator.

**FIGURE 5:**
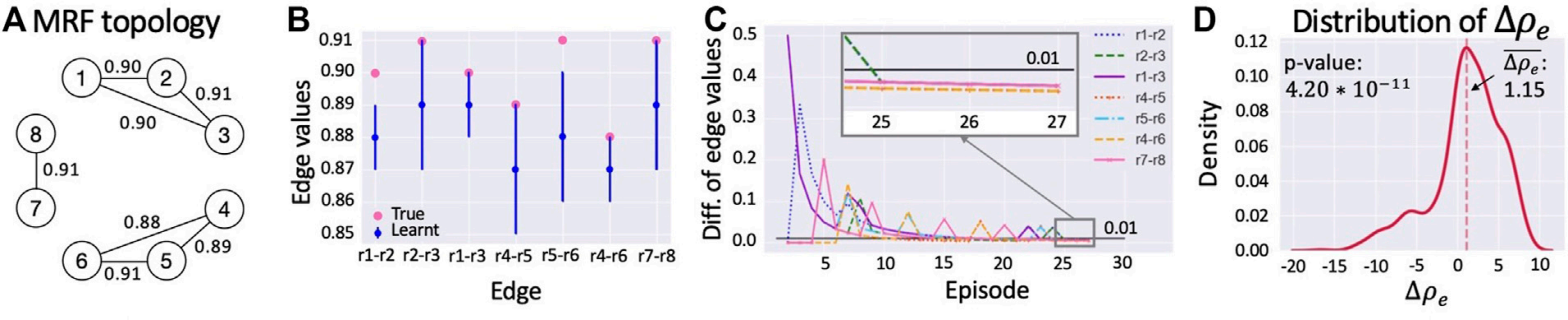
**(A)** True MRF topology with the equality probability constraints on its edges. **(B)** True MRF and average of the learned MRFs. Pink dots represent the values on the edges of the true MRF, whereas blue dots and lines correspond to the average edge values of the learned MRF and their standard deviations, respectively. **(C)** Difference of edge probability values during execution of the learning process until the convergence is reached in episode 27. The black line represents the convergence threshold. **(D)** The density of difference in discounted return from STD.

In this test, we first select a true MRF (i.e., a set of relationships among rock values; see Figure 5A). Edge probabilities are always set to 0.9 in the true MRF. We perform *NR = 10* runs. Hence, we compute 10 MRFs. In each run, we start preparing an empty MRF with the same topology (i.e., set of edges) as the true one (notice that our current method does not learn the topology of the MRF but only the potentials of an MRF with pre-defined topology). We learn the MRF potentials for several episodes determined by the stopping criterion with threshold *η* = 0.01 and *ce* = 3. The configuration of rock values changes with each episode satisfying the distribution defined by the true MRF. Then, we evaluate the performance of the learned MRF performing *NE = 100* episodes with EXT and STD algorithms, comparing the discounted return of each episode and averaging it over all the runs. In each episode, the agent performs *NS = 60* steps. The POMCP always uses 100,000 particles and performs the same number of simulations.

To prove that the introduction of the learned MRF provides a statistically significant improvement with respect to STD, we show that the average difference $\bar{\Delta \rho_{e}}$ between the discounted return obtained with EXT and the discounted return obtained with STD is significantly larger than zero. Notice that the difference is computed across all the NE = 100 episodes of each run (i.e., over 1,000 episodes in total). More precisely, at episode *e*, we compute the difference of discounted return *ρ*_*e*_ as $\Delta \rho_{e}=\rho_{e}^{EXT}-\rho_{e}^{STD}$ . Then, we compute the average of these values over all the episodes of all the runs *average discounted return* $\bar{\Delta \rho_{e}}$ .

#### 6.3.2 Results

The results we obtained using the C++ simulator are summarized in Figure 5. The main result is represented by the average difference in discounted return, $\bar{\Delta \rho_{e}}$ , achieved using the learned MRF in EXT with respect to STD that does not use any kind of prior knowledge. The value of $\bar{\Delta \rho_{e}}$ is 1.15 and corresponds to a performance improvement of 5.99% (Figure 5D). The distribution and corresponding average difference is computed over 100 episodes and 10 runs. To verify that $\bar{\Delta \rho_{e}}$ is statistically different from zero, we perform the Student’s *t*-test that confirms the statistical significance of the result as the *p*-value is lower than 0.05.

To explain the motivation for this improvement in Figure 5B, we compare the true and the learned MRF. On the *x*-axis, we display the edges of the MRF topology, whereas, on the *y*-axis, we show edge probability values. With pink dots, we represent the values on the true MRF edges (i.e., that from which we sampled the state-variable configurations of the learning episodes), whereas blue dots and lines represent, respectively, the average values of the learned MRF and their standard deviations (where the average is computed over the 10 runs performed in the learning process). The picture shows that the similarity between learned MRFs and the true one is very high. Moreover, Figure 5C depicts the trend of difference in probability values of all edges during a run of the learning process until it is stopped by the proposed criterion. In episode 25, on the *x*-axis, the difference in equality probabilities of all edges starts to be lower than 0.01, the threshold used in the stopping criterion. Since this condition persists in the next three episodes, the stopping criterion ends the learning phase in episode 27. Similar results with a different stopping criterion have been presented by Zuccotto et al. (2022).

### 6.4 Tests on the ROS Architecture for MRF Learning

In this section, we test the ROS architecture for MRF learning and present the results of our empirical analysis performed using this architecture.

#### 6.4.1 Experimental Setting

We perform tests using the MRF learning algorithm (Section 5.1) with the stopping criterion (Section 5.2) to learn the MRF on the ROS architecture proposed in Section 5.3. We perform our tests on the open-source multi-robot simulator Gazebo (Koenig and Howard, 2004), in which TurtleBot3 acts in the rocksample domain described in Section 6.1.1.

**FIGURE 6:**
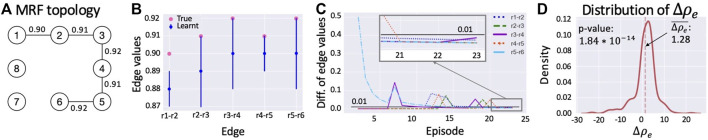
**(A)** True MRF topology with the equality probability constraints on its edges. **(B)** True MRF and average of the learned MRFs. Pink dots represent the values on the edges of the true MRF, whereas blue dots and lines correspond to the average edge values of the learned MRF and their standard deviations, respectively. **(C)** Difference of edge probability values during execution of the learning process until the convergence is reached in episode 23. The black line represents the convergence threshold. **(D)** Density of difference in discounted return from STD.

In this test, we first select a true MRF (Figure 6A). Edge probabilities are always set to the values on the edges of the true MRF topology. We perform *NR = 10* runs. In each run, we start preparing an empty MRF with the same topology as the true one. We learn the MRF potentials on the Gazebo environment, running the learning algorithm for several episodes determined by the stopping criterion with threshold *η* = 0.01 and *ce* = 3. The configuration of rock values changes in each episode, satisfying the distribution defined by the true MRF shown in Figure 6A. Then, we test the performance of the learned MRF performing *NE = 100* episodes with EXT and STD, comparing the discounted return of each episode and averaging it over all the runs. The MRF we used is the average of the 10 MRFs obtained during the learning process. In each episode, the agent performs *NS = 60* steps. The POMCP always uses *NP = 100,000* particles and performs the same number of simulations.

To prove that the introduction of the learned MRF provides a statistically significant improvement with respect to STD, we show that the average difference $\bar{\Delta \rho_{e}}$ between the discounted return obtained with the MRF learned with the ROS-architecture on Gazebo and the discounted return obtained with STD on the same framework is significantly larger than zero. Notice that the difference is computed episode by episode, and the average is computed across all the NE = 100 episodes of each run (i.e., over 1,000 episodes in total). More precisely, at episode *e*, we compute the difference of discounted return *ρ*_*e*_ as $\Delta \rho_{e}=\rho_{e}^{EXT}-\rho_{e}^{STD}$ . Then, we compute the average of these values over all the episodes of all the runs *average discounted return* $\bar{\Delta \rho_{e}}$ .

#### 6.4.2 Results

The results we obtained using the Gazebo simulator are summarized in Figure 6. The main result consists of the average difference of discounted return, $\bar{\Delta \rho_{e}}$ , achieved using the learned MRF in EXT with respect to STD that does not use any kind of prior knowledge. The value of $\bar{\Delta \rho_{e}}$ is 1.28 and corresponds to a performance improvement of 5.88% (Figure 6D). The distribution and corresponding average difference is computed over 100 episodes and 10 runs. To verify that $\bar{\Delta \rho_{e}}$ is statistically different from zero, we perform the Student’s *t*-test that confirms the statistical significance of the result as the *p*-value is lower than 0.05.

What allows this improvement is visible in Figure 6B in which we compare the true and the learned MRF. On the *x*-axis, we display the edges of the MRF topology, while on the *y*-axis, we show edge probability values. Pink dots represent the values on the true MRF edges (i.e., that from which we sampled the state-variable configurations of the learning episodes), whereas blue dots and lines represent, respectively, the average values of the learned MRF and their standard deviations (where the average is computed over the 10 runs performed in the learning process). The picture shows that the learned MRFs are very similar to the true one. Moreover, this also shows that using the proposed learning approach implemented in the ROS architecture allows us to learn accurate MRFs. Figure 6C depicts the trend of difference in probability values of all edges during a run of the learning process until it is stopped by the proposed criterion. In episode 20, on the *x*-axis, the difference in equality probabilities of all edges starts to be lower than 0.01, the threshold used in the stopping criterion. Because this condition persists in the next three episodes, the stopping criterion ends the learning phase in episode 23.

**FIGURE 7:**
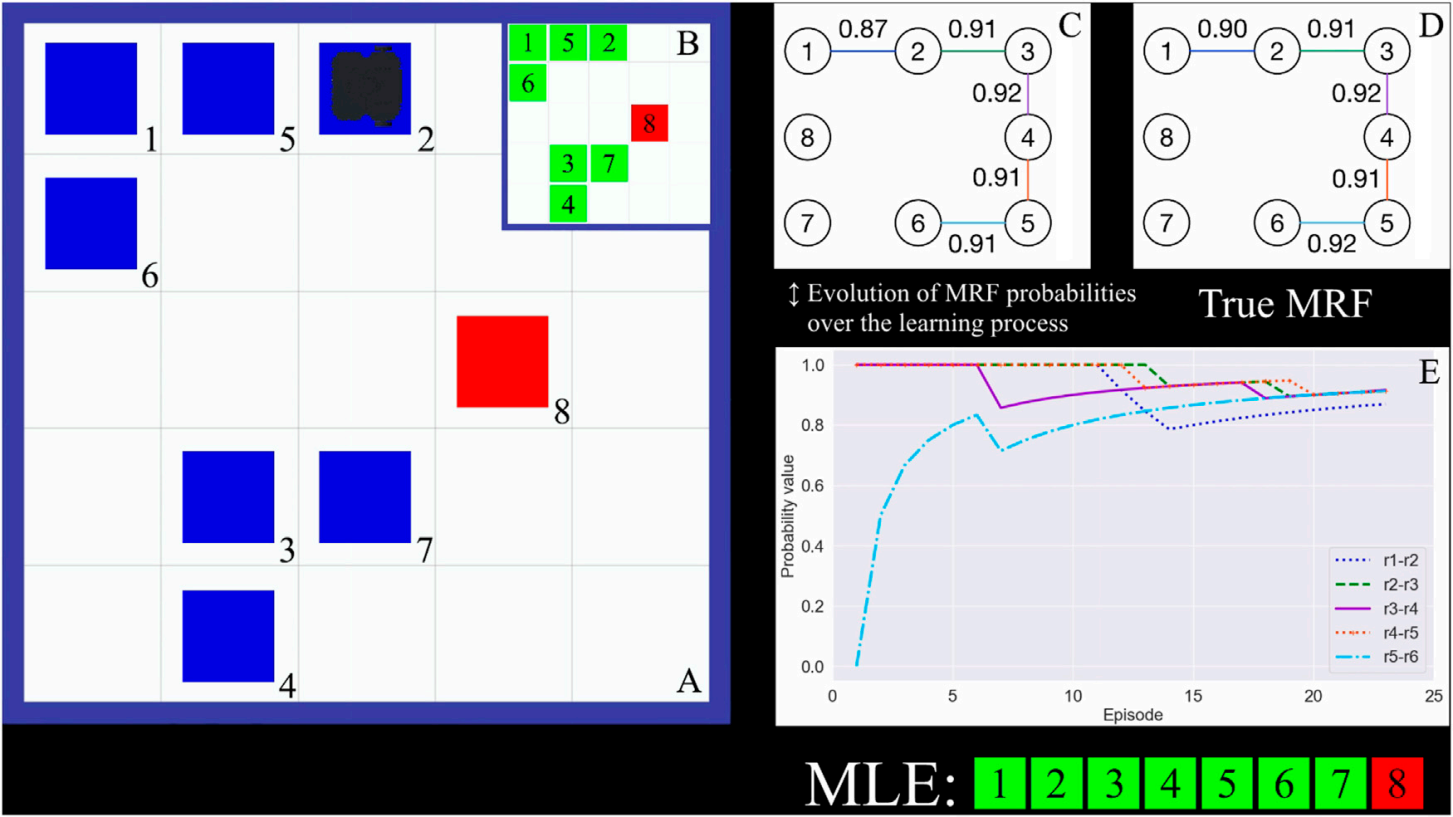
**(A)** Instance of the rocksample environment in which the TurtleBot acts during episode 23. **(B)** True rock values in episode 23. **(C)** MRF learned after 23 episodes. **(D)** True MRF, the one to be learned. **(E)** Evolution of edge probability values at the end of episode 23. When they converge, the learning process is ended.

To further clarify the learning process performed on the ROS-based architecture, we provide a video showing four learning episodes performed by a TurtleBot in the Gazebo simulator of the rocksample domain. Figure 7 shows a snapshot of the video. The mobile robot acting in the Gazebo environment is shown in Figure 7A. When it performs a sensing action on a rock, a question mark appears in the cell containing the rock. This cell becomes green or red if the outcome of the sensing action identifies the rock as valuable or valueless. When the agent performs a sampling action on a rock, the cell in which the rock is posed turns blue to specify that the rock has been collected. Figure 7B shows the true rock value configuration of the episode that satisfies the distribution defined by the true MRF. In Figures 7C,D, we show the edge probability values of the learned MRF updated at the end of episode 23 and the ones of the true MRF we aim at learning. In Figure 7E, we show the evolution of the edge probability values in the learned MRF, and when all the values reach the convergence in Figure 7E, the learning process ends.

### 6.5 Test on MRF Adaptation

We introduce the experimental setting used in our tests on ADA and then present the results of our empirical analysis.

#### 6.5.1 Experimental Setting

We perform two tests to evaluate the ADA method described in Section 5.4: one is performed on rocksample (5,8) and the second on velocity regulation. In both cases, a C++ simulator of the environment has been used to avoid the slowdown introduced by Gazebo because the physics of the environment is not fundamental to evaluating this algorithm. The goal of our tests is to highlight that by using ADA, we can, on average, improve the performance of the planner over both STD and EXT. This improvement is achieved by limiting the performance decrease generated when the learned or given by expert MRF is used on episodes characterized by unlikely state-variable configurations. In both tests, we perform *NR = 10* runs using an MRF that reflects probabilistic equality constraints among state-variable values learned using the MRF learning method of Section 5.1. To evaluate the performance of ADA, we perform *NE = 100* episodes using the MRF adaptation approach every time a discrepancy is detected during an episode and *NE = 100* episodes using the EXT algorithm. Then, we compare the discounted return of the two methods considering only the episodes in which the MRF adaptation approach has been used and average it over all the runs. In each episode, the agent performs *NS = 60* steps in the rocksample domain, whereas in the velocity regulation environment, it performs for *NS = 32* steps, namely, the number of subsegments in the path. The configuration of rock values (for the test on rocksample) and segment difficulties (for the test on velocity regulation) changes with each episode satisfying the distribution defined by the true MRF. The POMCP always uses *NP = 100,000* particles and performs the same number of simulations. We summarize the parameters used in our tests in Table 4.

**TABLE 4:** Parameters of tests on ADA.

Environment	NR	NE	NS	NP
Rocksample	10	100	60	100,000
Velocity regulation	10	100	32	100,000

To prove that the use of the MRF adaptation method in POMCP provides a statistically significant improvement with respect to the use of the MRF without adaptation, we show that the average difference $\bar{\Delta \rho_{e}}$ between the discounted return obtained with ADA and the discounted return obtained with EXT is significantly larger than zero. Notice that the difference is computed episode by episode, and the average is computed across all the episodes of each run in which ADA is used. More precisely, at episode *e*, we compute the difference of discounted return as $\Delta \rho_{e}=\rho_{e}^{ADA}-\rho_{e}^{EXT}$ . Then, we compute the average of these values over all the episodes of all the runs *average discounted return* $\bar{\Delta \rho_{e}}$ .

#### 6.5.2 Results

**FIGURE 8:**
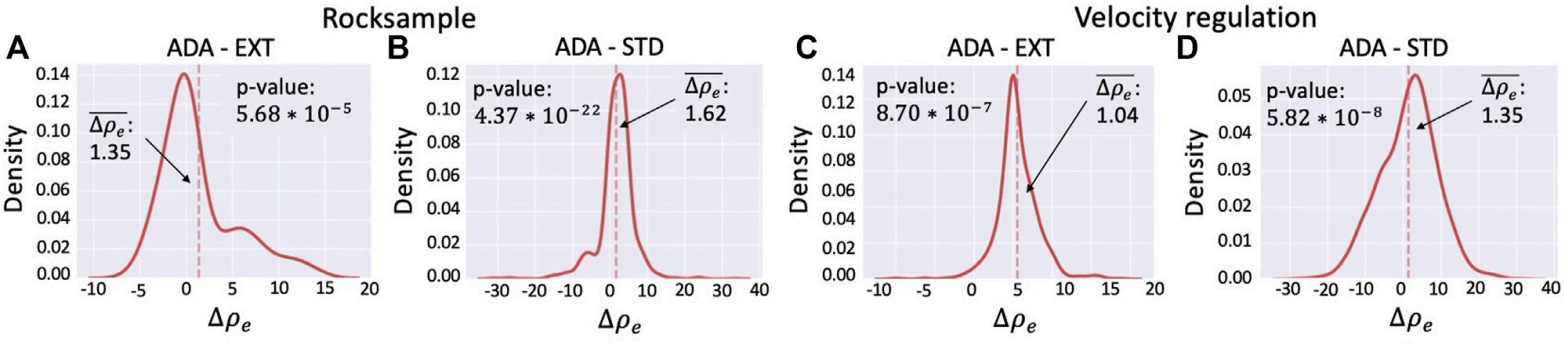
Density of difference in discounted return between **(A)** ADA and EXT on rocksample. **(B)** ADA and STD on rocksample. **(C)** ADA and EXT on velocity regulation. **(D)** ADA and STD on velocity regulation.

Figure 8 and Table 5 summarize the results of the two environments.

**TABLE 5:** Performance of ADA.

Environment	Comparison	$\bar{\Delta \rho_{e}}\left(\bar{\Delta \rho_{e}}\%\right)$	*p*-value
Rocksample	ADA—EXT	1.35 (6.54%)	5.68 × 10^–5^
	ADA—STD	1.62 (7.46%)	4.37 × 10^–22^
Velocity regulation	ADA—EXT	1.04 (3.51%)	8.70 × 10^–7^
	ADA—STD	1.35 (3.34%)	5.82 × 10^–8^

##### 6.5.2.1 Rocksample

Figure 8A shows the distribution of the differences between the discounted returns obtained using ADA and those obtained using EXT. The distribution is computed considering the 162 episodes (out of 1,000, i.e., 100 episodes for 10 runs) in which the adaptation mechanism was activated (i.e., at least a discrepancy between the learned MRF and the true state has been detected). The average distance is $\bar{\Delta \rho_{e}}=1.35$ , which corresponds to a 6.54% improvement (see the first line of Table 5). The *p*-value of the Student’s *t*-test guarantees that this average is significantly different from zero (Table 5). Figure 8B shows the distribution of the differences between the discounted returns obtained using ADA and those obtained using STD. This distribution is computed on 1,000 values (i.e., 100 episodes for 10 runs). The average is $\bar{\Delta \rho_{e}}=1.62$ , which corresponds to a 7.46% improvement (see the second line of Table 5). Also, in this case, the *p*-value of the Student’s *t*-test guarantees that this average is significantly different from zero (Table 5). Therefore, we can state that the improvement is, on average, statistically significant.

##### 6.5.2.2 Velocity Regulation

The experiments performed on the velocity regulation domain confirm the positive results obtained on the rocksample. Figure 8C shows the distribution of the differences between the discounted returns obtained using ADA and the ones obtained using EXT. The distribution is computed considering 714 episodes (out of 1,000), that is, the number of episodes in which the adaptation mechanism was activated. The average difference is $\bar{\Delta \rho_{e}}=1.04$ , corresponding to a 3.51% performance improvement (third line of Table 5). This average is significantly different from zero because the *p*-value of the Student’s *t*-test is lower than 0.05 (Table 5). Figure 8D shows the distribution of the differences between the discounted return obtained with ADA and the ones obtained with STD. In this case, the distribution is computed on 1,000 values (i.e., all the 100 episodes for all the 10 runs). The average $\bar{\Delta \rho_{e}}=1.35$ and it corresponds to an improvement of 3.34% (fourth line of Table 5). Furthermore, in this case, the *p*-value of the Student’s *t*-test guarantees that the value of $\bar{\Delta \rho_{e}}$ is statistically different from zero (Table 5). Thus, the performance improvement obtained is, on average, statistically significant.

**FIGURE 9:**
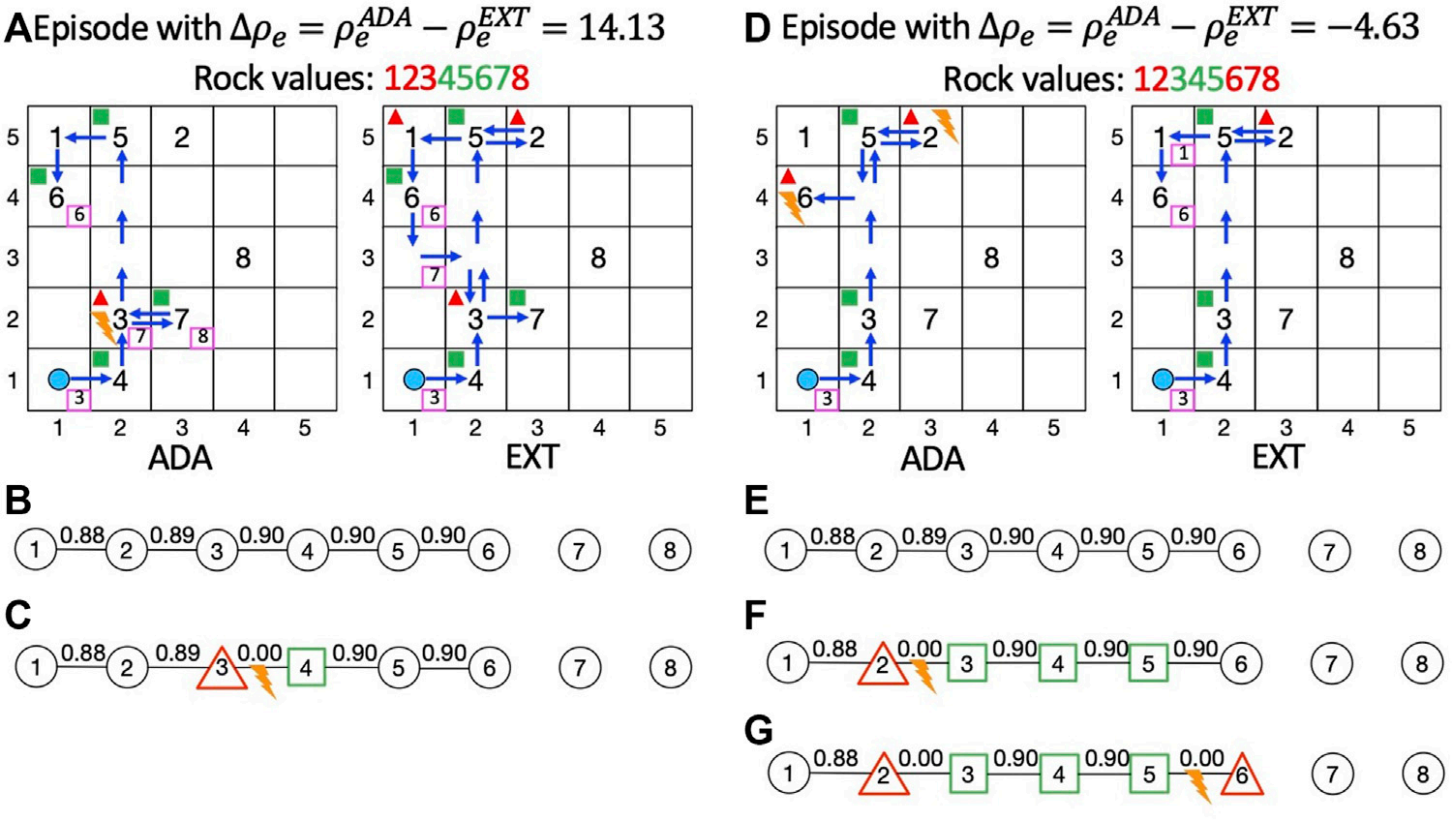
**(A)** Relevant actions of the execution traces of an episode with a *positive* Δ*ρ*_*e*_ between ADA (on the left) and EXT (on the right). **(B)** Initial MRFs (learnt). **(C)** Adapted MRF, P(3,4)=0 . **(D)** Relevant actions of the execution traces of an episode with a *negative* Δ*ρ*_*e*_ between ADA (on the left) and the use of EXT (on the right). **(E)** Initial MRFs (learnt). **(F)** MRF after the first adaptation, P(2,3)=0 . **(G)** MRF after the second adaptation, P(5,6)=0 .

Finally, to highlight the different behavior of ADA compared to EXT, in Figure 9A, we show the behavior of ADA (on the left) and EXT (on the right) in a specific episode in which ADA is used and gives a performance improvement. Instead, in Figure 9D, we show the behavior of a specific episode in which the use of ADA yields a decrease in performance. In each figure, we represent on the left grid the actions performed by the agent using ADA, whereas, on the right grid, we represent its action using the learned MRF. To denote the presence of a rock in a specific cell, we use its ID (from 1 to 8). The agent’s starting position is represented by the light blue circle, and blue arrows indicate the path traveled by the agent. In pink-bordered boxes, we indicate the ID of the rock that the agent senses from a cell. With green boxes and red triangles we, respectively, represent the fact that the agent samples a valuable or valueless rock in the corresponding cell. Finally, the orange lightning means that a discrepancy is detected and that the adaptation approach is used as previously described in Section 5.4.

Figure 9A shows lighting in cell (2,2) of the left grid. The learned MRF (Figure 9B) expresses a high equality probability between rock 4 and rock 3 (P(3,4)=0.90) . Thus, after the valuable rock 4 has been collected, the agent is encouraged to also sample rock 3. In the true state-variable configuration, instead, rock 3 is valueless; thus, a discrepancy with the learned MRF is detected. Then, the probability value on the edge P(3,4) is set to 0 because the assignments of rock 3 and 4 are different (Figure 9C). Afterward, the particle filter is re-initialized according to Algorithm 2 and the belief re-computed. The positive effect of the proposed method is clearly visible because the agent does not sample rocks 1 and 2. Rock 2, in fact, is related to (valueless) rock 3 by an equality probability of 0.89 (on average) in the learned MRF, so the agent is not encouraged to sample rock 2 (Figure 9C). Rock 1, in turn, is related to rock 2 by P(1,2)=0.88 ; thus, the agent does not sample rock 1. For the same reason, rocks 5 and 6 are sampled due to the equality probability that relates the assignment of rock 5 to the valuable rock 4 and the one that relates the assignment of rock 5 to the value of rock 6 (both probabilities are 0.9 on average). On the right grid of Figure 9A, instead, we see what happens when the learned MRF, despite its correctness in probabilistic terms, does not reflect the state-variable configuration in the specific episode at hand. The agent samples the valueless rock 3 then; because its knowledge about the environment does not change, the agent also samples rocks 1 and 2, both valueless. In this episode, ADA allows limiting the negative effect of a misleading MRF obtaining a Δ*ρ*_*e*_ of 14.13.

In Figure 9D, instead, we depict the most relevant agent actions of an episode in which ADA performs worse than EXT. On the left grid, we show that two discrepancies with the learned MRF (Figure 9E) are detected, respectively, in cell (2,5) and (1,4). Thus, ADA is used twice in this episode. The effect of the first usage of ADA (Figure 9F) consists of discouraging the agent from sampling rock 1, whereas the second (Figure 9G) does not influence any other sampling action because rock 5 has already been sampled and no other state variable has equality relationships with rock 6. In the right grid, the agent performs a sensing action on rock 6 that returns a negative response discouraging the agent from sampling the rock. The different behavior of the agents about rock 6 gives a negative value for Δ*ρ*_*e*_, which is −4.63.

## 7 Conclusion and Future Work

We presented three main contributions to the literature: a methodology for learning state-variable relationships in POMCP in the form of an MRF, an algorithm for adapting the MRF to the true states encountered while using the MRF in POMCP, and a ROS-based architecture that allows running the MRF learning and the POMCP with the MRF on real robotic platforms. Results show that the MRF adaptation algorithm achieves a statistically significant performance improvement over the use of the MRF without adaptation. Moreover, using the proposed architecture, we managed to learn informative MRFs that yield statistically significant performance improvement over standard POMCP. Our future work will focus on two main directions. From a methodological point of view, an interesting problem concerns integrating the learning process into the context of information gain problems on POMDPs. The goal, in that case, is to tune the exploration-exploitation trade-off considering the learning of the MRF. From an application viewpoint, we aim to extend the proposed ROS architecture to support other kinds of platforms, such as robotic manipulators, to assess our method on different problems that can be formalized as POMDPs.

## Data Availability

Code availability: https://github.com/kriato/pomcp_mrf_ros. The original contributions presented in the study are included in the article/Supplementary Material. Further inquiries can be directed to the corresponding authors.

## Footnotes

1
https://github.com/kriato/pomcp_mrf_ros
